# The “Macro” World of microRNAs in Hepatocellular Carcinoma

**DOI:** 10.3389/fonc.2015.00068

**Published:** 2015-03-25

**Authors:** Kaveri Sidhu, Neetu Rohit Kapoor, Vijaya Pandey, Vijay Kumar

**Affiliations:** ^1^Virology Group, International Centre for Genetic Engineering and Biotechnology (ICGEB), New Delhi, India

**Keywords:** hepatitis B virus, hepatitis C virus, hepatocellular carcinoma, microRNA, onco-miR

## Abstract

Hepatotropic viruses such as hepatitis B virus (HBV) and hepatitis C virus (HCV) are the major etiological agents associated with development of hepatocellular carcinoma (HCC). Progression of HCC is a multistep process that requires sequential or parallel deregulation of oncogenic and tumor suppressive pathways leading to chromosomal instability and neoplastic phenotype. In the recent years, microRNAs (miRNAs) have carved their own niche alongside oncogenes and tumor suppressors, owing to their innate ability to receive and relay multiple signals. Not surprisingly, miRNAs are fast emerging as central player in myriads of malignancies including HCC. miRNAs are reported to participate in initiation and progression of HCC, and have also been clinically correlated with risk assessment, disease grade, aggressiveness, and prognosis. Despite extensive data available on the role of miRNAs in HCC, there is a pressing need to integrate and evaluate these datasets to find its correlation, if any, with causal agents in order to devise novel interventional modalities. Through this review, we attempt to bridge the gap by consolidating the current knowledge and concepts in the field of HCC-related miRNAs with special emphasis on HBV and HCV. Further, we assess the potential of common as well as unique signatures that may be useful in developing novel biomarkers and therapeutics.

## Introduction

According to a WHO estimate, hepatitis B virus (HBV) and hepatitis C virus (HCV) together account for ~78% of hepatocellular carcinoma (HCC) incidence worldwide and are the second leading cause of cancer mortality (1). High morbidity observed in HCC is majorly attributed to lack of early detection markers and poor prognosis, which limit the options for chemotherapy, adjuvant therapies, or surgical procedures (2). Hence, exploration of novel frontiers in HCC diagnosis and therapeutics remain high priority research areas.

Recent studies suggest an indispensable role played by microRNAs (miRNAs) in tumor growth, and immune evasion. miRNAs constitute a major class of well-conserved, small non-coding RNAs that can up- or down-regulate gene expression (2, 3). Accordingly, miRNAs can function both as tumor suppressor (TS-miR) and oncogene (onco-miR) impacting both pro- and anti-proliferative cascades (4). To date, nearly 2000 different miRNAs have been identified in humans. Stability of miRNAs in blood circulation makes them ideal candidate for use in diagnosis and treatment of cancers. Further, tumor miRNA profiles can be helpful in defining relevant subtypes, patient survival, treatment response, and risk prediction (5). Given the vast repertoire of cancer-related pathways regulated by miRNAs, this class of biomolecules appears to be a driving force for oncogenesis as well as “Achilles’ heel” for therapeutic targeting. The intricate processes of miRNA biogenesis and maturation are easy targets of stealth hepatitis viruses (6). In the next section, we overview how these processes are modulated in HCC.

## Transcriptional Regulation of miRNA

The transcription of miRNA is guided by RNA polymerase (pol II) regulators, which are often deregulated in case of liver pathologies. In HBV- and HCV-associated pathogenesis, c-Myc along with viral oncoproteins modulates miRNA expression, to create an oncogenic milieu (7) facilitating the binding of transcriptional repressor complexes to miRNA promoters to allow its sustained expression (8, 9). For example, c-Myc binding to miR-122 promoter (a liver-specific TS-miR) prevents RNA pol II recruitment and H3K9 acetylation (10). c-Myc also down-regulates HNF3β, a liver-specific transcription factor, involved in transcription of miR-122. Since c-Myc itself is a target of certain miRNAs, the transcriptional control of its regulators sets on a positive feedback loop for c-Myc expression in HCC (8, 11).

c-Myc is also reported to stimulate DROSHA promoter and thus, indirectly regulate the stability of DiGeorge syndrome critical region gene 8 mRNA (12). Although c-Myc normally functions as a negative regulator of miRNAs, the levels of onco-miRs like miR-21 and miR-17-92 polycistron are elevated in c-Myc microenvironment, which down-regulate tumor suppressors (13). HCV infection *per se* can cause down-regulation of TS-miR-181c by promoting the recruitment of CCAAT/enhancer binding protein β (C/EBP-β) (14). In contrast, HBV positively regulates miR-181a transcription – an index of poor survival whereas it negatively regulates tumor suppressors, WIF1 and DKK3 (controllers of Wnt signaling pathway and miR181a targets) possibly via miR181a (15–17). Inactivation of p53 in HCC leads to down-regulation of its transcriptional targets such as miR-34, miR-200, and miR-15/16, which allows cell proliferation and metastasis (18). p53 inactivation also alters miRNA biogenesis either by directly binding to DROSHA or via down-regulation of DICER-1 (19), which may define miRNA target genes in HCC.

## Post-Transcriptional Regulation of miRNA

Hepatitis viruses often deregulate pro-proliferative pathways in HCC affecting phase-specific control of cyclins by miRNAs (6). Impairment of DICER-1 is also frequently observed in HCC combined with tumor stemness (20). Post-transcriptional regulators of Argonaute (Ago-1 and Ago-2) such as lin-41 are over-expressed in HCC in a c-Myc dependent fashion, which down-regulates Ago protein (21). Whereas low levels of miR-99 and miR-199a induce Ago-2 expression with consequent increase in miR-21 (22, 23). Modulation of TGFβ signaling in HCC also involves miRNAs to promote tumorigenesis (24, 25). Interestingly, TGFβ effector SMAD1/5 along with RNA helicase p68 increases the maturation rate of miR-21 and miR-199 contributing to vascularization (25).

## Epigenetic Alterations of miRNA

Concordant hyper methylation of miRNA genes is frequently seen in HCC (26, 27). The master controllers of proliferation, like c-Met are epigenetically silenced by TS-miRs (28). An auto-feedback loop of hyper-methylation and gene suppression is suggested for miR-148a and miR-152 in HCC (29, 30). Acetylation status at the pri-miRNA promoters can also suppress miRNA and help relieve the suppressive effect of TS-miRs (31). Besides, sulfated glycolipids can facilitate intrahepatic metastasis (32).

HBV and HCV oncoproteins may also regulate miRNA expression (33) by engaging DNA methyltransferases (DNMT), which cause global hyper-methylation (34, 35). The HBx oncoprotein is known to sequester the epigenetic modifier PPARγ in order to down-regulate miR-122 in HCC (36). The HBx-mediated epigenetic silencing of miR-205 stabilizes HBx mRNA, and aggravates disease (37). Similarly, HCV core protein can down-regulate miR-124 and miR-345 levels by inducing DNMT expression and abrogating apoptosis (38).

## Single Nucleotide Polymorphism and Genetic Alterations

Single nucleotide polymorphism (SNP) in the regulatory or coding regions of miRNA is essential for onco-miR expression or silencing of TS-miRs in HCC. TS-miRs such as miR-34b/c are poorly expressed in HCC owing to SNP RS4938723, which inhibits the recruitment of transcription factor GATA (39). Alternately, SNPs in the coding region of miR-196a2 and promoter of miR-106b-25 up-regulate their expression and contribute toward HCC (40, 41). SNP in the stem–loop of pre-miR-146a affects its processing efficiency and increases the risk of HCC (42). As miRNA genes are often located close to fragile sites (43), their translocation is frequently observed in many cancers. The translocation of 5′ end of the *hcr* gene (encoding miR-122) to *c-myc* locus can cause a massive 50-fold increase in c-Myc expression and a consequent down-regulation of miR-122 in woodchuck hepatitis virus-related HCC (24, 44).

Thus, miRNAs have emerged as crucial players in virus–host interactions, where hepatitis viruses can alter miRNA biogenesis, which in turn control key cellular pathways to establish a successful infection as discussed in the next section.

## Deregulation of miRNA in HCC

**Figure 1:**
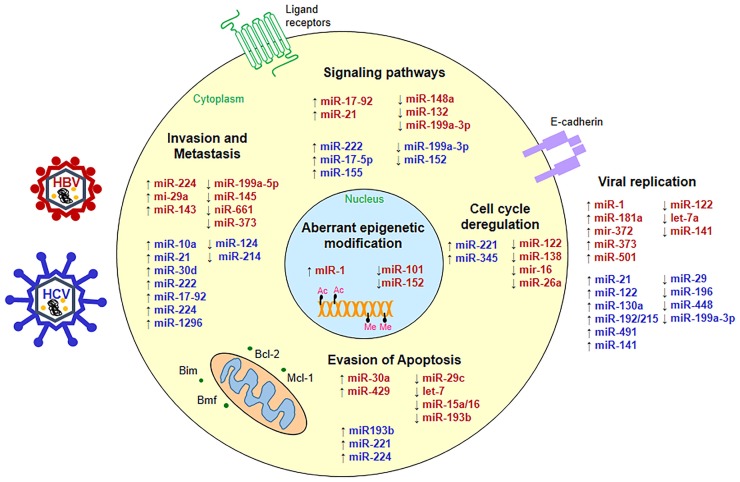
**Status of micro-RNAs during hepatitis virus infection**. miRNAs are up- (↑) or down-regulated (↓) in HBV- (red) or HCV-associated HCC (blue) and interfere with key cellular pathways such as cell signaling, apoptosis, cell cycle, metastasis as well as viral replication to promote hepatocarcinogenesis.

Deregulated miRNAs play a pivotal role in supporting viral replication and perturbing key cellular processes in HBV- and HCV-associated HCC as depicted in Figure 1.

## miRNA in the Regulation of Viral Replication

HBV and HCV engage host miRNAs to complete virus life cycle and establish chronic infection. For example, miR-122, which is often down-regulated in many liver pathologies, shows opposing effects on HBV and HCV replication (45). miR-122 restricts HBV replication by targeting cyclin G1 and allowing p53-mediated suppression of viral genes (46). Paradoxically, miR-122 may support HBV replication by down-regulating heme oxygenase-1 (HO-1), a negative regulator of HBV transcription (47). Further, miR-1 and miR-372 along with miR-373 can enhance HBV replication by activating farnesoid X receptor-alpha (FXRA) and nuclear factor I/B, respectively (48, 49). Interestingly, miR-15b can also promote viral replication by binding to hepatocyte nuclear factor 1α and inducing HBV enhancer 1, while down-regulated miR-15b levels lower virus load and allow persistent HBV infection (50).

In case of HCV, however, binding of miR-122 to the internal ribosome entry site (IRES) in the 5′-UTR of viral RNA augments viral replication and promotes HCV translation in an Ago-2-dependent manner (45, 51). miR-122 and miR-196 also target HO-1 by differentially regulating its repressor Bach-1 to complement or inhibit HCV replication, respectively (52, 53). Akin to miR-122, miR-199a-3p also binds to the IRES element and inhibits HCV replication (53). Interestingly, miR-27a is over-expressed in HCV cases which lowers the viral load to escape immune surveillance through a negative feedback loop (54).

## Role of Deregulated miRNAs in Key Cellular Processes

The down-regulation of TS-miRs and concomitant elevation of onco-miRs also affect some key cellular processes, which support HCC.

### Cell cycle and proliferation

microRNAs can target cell cycle regulators to override some checkpoints and support uncontrolled proliferation. For example, TS-miR-26a targets cyclin D2/E2 that control G1/S boundary of the cell cycle since its re-expression prevented disease progression in a pre-clinical HCC model (55). Further, down-regulated levels of miR-195 and miR-138 act as tumor suppressor by targeting cyclin D1 and cyclin D3 (56, 57), while onco-miRs miR-221 and miR-222 promote tumor growth by down-regulating p27, p57, and DNA damage inducible transcript 4 (58). The let-7 family of miRNAs, which is down-regulated in HCC, allows cell proliferation by negatively regulating STAT3 and c-Myc levels and positively regulating p16^INK4A^ (59, 60). MiR-99a suppresses G1 arrest of cancer cells by targeting cyclin D1 via mTOR (61).

### Suppression of apoptosis

Cancer cells evade apoptosis by perturbing the balance between pro and anti-apoptotic factors. In aggressive HCC cases, miR-25 and miR-221 are over-expressed, which target pro-apoptotic proteins Bim and Bmf, respectively (62, 63). Down-regulated miR-29, miR-101, and miR-122 relieve suppression of their anti-apoptotic targets Bcl-2, Mcl-1, and Bcl-w, respectively, leading to increased survival in HCC (64). HBx oncoprotein down-regulates let-7 and miR-15a/16 leading to increased anti-apoptotic activity (65). Similarly, low levels of TS-miR-29c in HCC target tumor necrosis factor alpha-induced protein 3 to prevent apoptosis (66).

### Alteration of signaling pathways

Tumor suppressor PTEN, a negative regulator of PI3K/AKT signaling is frequently targeted by elevated levels of miR-21, miR-222, and miR-29a in HCC (59, 64). miR-199-3p targets mTOR and c-Met, a HGF receptor, which controls downstream PAK4/Raf/MEK/ERK pathway (67). Restoration of miR-199-3p levels in hepatoma cells leads to G1 arrest, enhanced susceptibility to hypoxia and drug (67). HBx suppresses the p53-dependent activation of miR-148a, resulting in upregulation of AKT, ERK, and activation of mTOR pathway, which promotes tumor growth and metastasis in a HCC mouse model (68). miR-17-5p, a member of onco-miR-17-92 cluster, activates p38-MAPK- and Hsp27 pathway to promote cell migration and proliferation (69). Further, miR-222 overexpression confers metastatic potential on cancer cells by activating Akt pathway (70). Besides its pro-inflammatory role, miR-155 upregulation by HCV results in increased nuclear accumulation of signal transducer β-catenin to promote HCC (71).

### Epithelial–mesenchymal transition and metastasis

Prior to metastasis, tumor cells undergo epithelial–mesenchymal transition (EMT) involving loss of E-cadherin, gain of vimentin, collagen I, and fibronectin. miR-224, a highly expressed miRNA in HCC, promotes tumor growth and metastasis by silencing its target genes Cdc42, CDH1, PAK2, and BCL-2 (72). Elevated levels of miR-29a and miR-148a, which target PTEN and miR-143 also down-regulate fibronectin type III domain-containing protein 3B to enhance hepatoma cell migration (73, 74). HBx engages miR-661 and miR-373 to stimulate metastasis associated-1 protein and suppress E-cadherin, respectively (75). TS-mir-34a and miR-125b that, respectively, target c-Met and LIN28B2 oncogenes are inversely correlated with metastasis in HCC (76, 77). In contrast, miR-200c suppresses EMT of liver cancer stem cells (CSCs) by upregulating E-cadherin, reducing vimentin, and inhibiting metastasis through repression of neurotrophic receptor tyrosine kinase 2 (78). Zeb1 and Zeb2 transcription factors are also targeted by miR-141/200c to alter E-cadherin and related gene expression involved in cell polarity (79). Not surprisingly, miR-141/200c cluster is often silenced in cancer by DNA methylation (80).

Thus, miRNAs can initiate multistep process of cancer by perturbing normal cell homeostasis and endowing cells with the ability to invade and metastasize. Further, the crosstalk between miRNAs could have a multiplier effect on their target genes as well as drug resistance cancer phenotype.

## miRNAs as Diagnostic Markers in HCC

**Figure 2:**
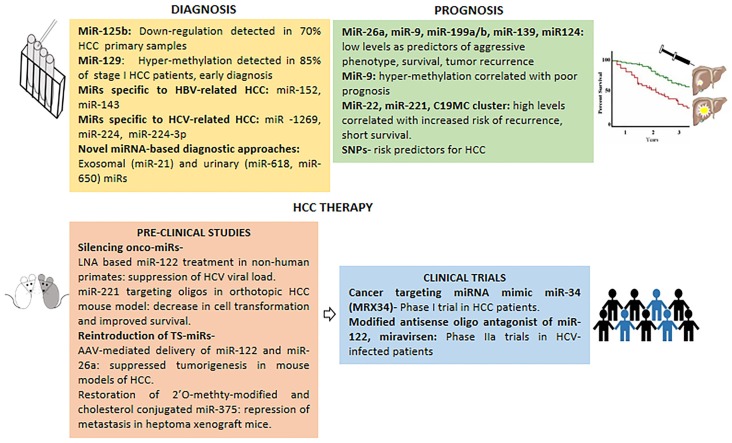
**Micro-RNAs in clinical management of HCC**. miRNAs with potential in HCC diagnosis, prognosis and therapy (pre-clinical studies and clinical trials).

The lack of effective diagnostic methods for early HCC has rendered the overall survival rate to a low 0–14% from the time of clinical diagnosis (81). Modest precision of currently used diagnostic markers such as alpha-fetoprotein (AFP) calls for testing the prospect of miRNAs as HCC biomarkers. Circulating miRNAs are highly stable in serum owed to their resistance to RNAse, extreme pH, and temperature, hence are perfect as biomarkers for detecting early stage, presymptomatic diseases such as HCC as depicted in (Figure 2).

The serum levels of miRNAs undergo alterations in HCC patients as evident from low levels of miR-16, miR-199a, and high levels of miR-21, miR-221, miR-222, miR-223, and miR-224 in serum samples (5). miR-125b is down-regulated in ~70% of primary HCC samples thus could be a good candidate for diagnosis (77). Further to expand the repertoire of prospective miRNAs in early diagnosis of HCC, down-regulated levels of TS-miR-129 in HCC can be detected in plasma samples from 85% of stage I HCC patients as compared to AFP in just 10% of stage I cases (82).

Detection of HBV- or HCV-positive HCC cases, especially those independent of cirrhosis etiology, poses a greater challenge due to lack of biomarkers. In light of this, unique expression profile of serum miRNAs in HBV- and HCV-positive HCC patients can serve as a fingerprint for distinguishing between HBV and HCV cases. Not surprisingly, miRNAs such as miR-1269, miR-224, and miR-224-3p are significantly altered specifically in HCV-associated HCCs (83). In contrast, miR-152 and miR-143 are aberrantly regulated in HBV-related HCC and hence constitute potential diagnostic markers for HBV-related HCC cases (84, 85).

Serum miRNAs enriched in exosomes can also serve as valuable non-invasive HCC biomarkers for both diagnostic and prognostic purposes. Indeed, recent findings have shown that serum exosomal miR-21 from HCC patients provides increased sensitivity of detection compared to whole serum (86). Interestingly, a report on urinary miRNAs such as miR-618 and miR-650 has opened the prospect to use them as biomarkers for early detection of HCV-induced HCC (87).

## Predictive Prognostic Value of miRNAs in HCC

It is being increasingly realized that miRNAs may possess an edge over mRNA as prognostic indicators owed to their stability in clinical samples and robust expression patterns. In fact, expression profiles of a panel of 20 miRNAs can be used as metastatic predictor, correlating with survival as well as relapse rates in HCC (88). As indicated in Figure 2, hyper-methylation of miRNA promoters seems to correlate with poor prognosis, exemplified by aberrant methylation of miR-9 and corroborates with clinical outcomes (89). Further, miR-199a/b and miR-139, which are frequently down-regulated in most HCC patients, show a significant correlation with poor survival (89, 90). Likewise, low expression of miR-124 in HCC seems to be associated with more aggressive behavior and shorter survival (89). While, high levels of miR-22, miR-221, and C19MC miRNA correlate with increased risk of tumor recurrence and shorter survival (59).

Interestingly, the patterns of miRNA expression as well as SNPs in some miRNAs seem potent as predictors of patient response to various therapeutic strategies as well as disease risk (63). In Chinese and Turkish populations, a positive association has been noticed between rs11614913 (C →T) SNP in miR-196a-2 and HCC susceptibility (91, 92). Similarly, “TTCA” insertion (rs3783553) in 3′-UTR of IL-α gene, which ablates the binding site for miR-122 and miR-378 leading to upregulation of IL1-α, correlates well with HCC development (93).

## miRNAs in HCC Therapy

Alterations in miRNA expression are frequently associated with HCC disease physiology; hence miRNAs could be used as potential druggable targets in HCC management. One of the approaches in miRNA-based HCC therapy involves using antagomirs against onco-miRs. Non-human primates chronically infected with HCV when treated with locked nucleic acid (LNA) specific for miR-122 (a positive regulator of HCV replication), exhibited long-term suppression of HCV viral load, supporting therapeutic use of miRNA in HCC (2). In another pre-clinical study, involving an orthotopic HCC mouse model, oligonucleotides targeting miR-221 inhibited cell transformation and improved survival, underscoring its potential in HCC therapeutics (5). Another potential therapeutic approach for HCC treatment entails restoration of TS-miRs, thus serving as anti-cancer agents. For instance, AAV-mediated delivery of miR-122 and miR-26a and systemic restoration of miR-124 can suppress tumorigenesis in animal models of HCC. Similarly, restoration of miR-375 (2′*O*-methyl-modified and cholesterol conjugated form) and miR-29 could inhibit tumorigenesis in pre-clinical HCC models (2, 5).

Delivery of miRNA mimics can also be used in HCC therapy. Indeed, a cancer-targeting miRNA mimic of miR-34 (MRX34) is in phase I clinical trial performed by Mirna Therapeutics Inc., in HCC patients (5, 94). Interestingly, the mimic miRNAs are delivered using Smarticles^®^, which are anionic at neural pH, but attain cationic charge in acidic tumor environment thus minimizing off-target effects (5). Importantly, the therapeutic potential of miR-122 antagonist, miravirsen was evident from a multi-centric phase IIA trial, which showed sequestration of mature miR-122 and reduction of viral load (95).

Though miRNAs possess tremendous therapeutic potential for HCC, a major concern remains their delivery system. miRNAs can be incorporated into PEG-gylated stable nucleic acid lipid particles (SNALPS), to extend the circulation time. Further, virus-like particle (VLP) dependent delivery gives the leverage of natural tissue tropism, albeit with risk of eliciting immune response (5).

The emergence of miRNAs as novel clinical biomarkers is set to change the face of HCC diagnosis and therapeutic procedures. The proposition of miRNA profiles serving as signatures to distinguish HCV from HBV cases, though awaiting clinical evaluation, offers the advantage of accurate diagnosis and appropriate therapeutic course.

## Conclusion

The heterogeneity in individual cases of cancer including HCC demands development of personalized medicine to ensure most effective treatment with minimal side effects. In such a scenario, unique tumor specific miRNA signatures, as reviewed here, will help design accurate diagnostics and therapeutics tailored to individual needs. The ease of delivering oligonucleotides to liver and high tolerance of normal liver cells to supplements of deficient miRNAs make HCC an ideal model to test miRNA therapy (2). Preliminary evidence in non-human primates indicates that miRNA-based treatment of chronic HCV infection provides prolonged alleviation of virus-induced liver pathology with a high barrier to viral resistance suggesting miRNAs may function as better antivirals than conventional drugs (2). CSCs, resistant to conventional chemotherapy and cause of cancer recurrence, form the major obstacle in successful treatment of the disease. The discovery of stemness associated miRNAs in liver cancer such as miR-181, miR-150, and miR-548c-5p warrants further evaluation of their potential as druggable targets in therapies against liver CSCs (96). The gender specific discrepancy in HCC incidence can be in part explained by microRNA signatures such as higher levels of TS miR-26a and miR-26b in females that may provide them a protective edge over males (97). Elevated levels of miR-18a in HCC female patients, which suppresses protective effects of estrogen by targeting estrogen receptor-alpha, can be used as a risk predictor of HCC development in female population (98). Finally, the quest for yet elusive HBV and HCV encoded miRNAs poses a challenge, which when overcome may help in devising novel strategies to silence viral miRNA and cure liver cancer.

## Conflict of Interest Statement

The authors declare that the research was conducted in the absence of any commercial or financial relationships that could be construed as a potential conflict of interest.
